# Engaging clergy and congregants as “lay-scientists” to promote community health and wellbeing in rural and urban Alaska: a community-engaged pilot study

**DOI:** 10.3389/fpubh.2026.1737078

**Published:** 2026-10-29

**Authors:** Nathan T. West, Megan Waddell, Diane K. King, Shirley T. Le, Sofia A. Portillo, Kelsey R. Day, Beverly R. Bateman, Undra Parker, Nico Reijns, Abby C. King

**Affiliations:** 1 Center for Behavioral Health Research and Services, University of Alaska Anchorage, Anchorage, AK, United States; 2 Department of Epidemiology & Population Health, Stanford University, Stanford, CA, United States; 3 Department of Public Health Sciences, University of Virginia, Charlottesville, VA, United States; 4 Nome Church of the Nazarene, Nome, AK, United States; 5 Shiloh Missionary Baptist Church, Anchorage, AK, United States; 6 Turnagain United Methodist Church, Anchorage, AK, United States; 7 Department of Medicine (Stanford Prevention Research Center), Stanford University School of Medicine, Palo Alto, CA, United States

**Keywords:** citizen science, community-based participatory action research, congregational health and wellbeing, faith-based health promotion, social and built environment

## Abstract

**Introduction:**

The globally implemented *Our Voice* citizen science method has addressed social and structural health-related barriers in many community settings. Currently, however, this participatory action research method has not been adapted and tested in faith-based settings, which are trusted and well positioned to reach communities for health promotion and chronic disease prevention. The purpose of this study is to describe our process for adapting and piloting *Our Voice* and report lessons that will meaningfully engage faith-based communities in health promotion.

**Methods:**

Prior to *Our Voice* implementation, we used a community-academic design approach and sought input and recommendations from faith community members to increase reach and ensure *Our Voice* content and procedures aligned with cultural traditions. We then recruited three churches in which to pilot *Our Voice* and collected key process data through a baseline survey (e.g., lay-scientist demographics, physical activity behavior) and community notes (e.g., meeting attendance, lay-scientist engagement) to inform refinement of procedures. Trained research staff conducted objective environmental audits using a validated church environmental audit tool to describe the health promotion environment of each setting. Trained teams of clergy and congregants i.e., “lay-scientists”, next conducted an *Our Voice* environmental assessment using the Discovery Tool mobile app to evaluate each participating church’s space, features and proximal built environments.

**Results:**

The lay-scientist teams collected a total of 216 geo-tagged photos with narratives which were used to identify church-specific themes and relevant action plans to address priority needs. Example themes and action plans include: 1) Implementing congregational health needs assessments (e.g., brief online surveys for the broader congregation); 2) displaying health messaging across the church building (e.g., physical activity and healthy eating recommendations); and 3) encouraging both congregation- and community-wide health promotion (e.g., church-sponsored health summits, gardening initiatives with local schools).

**Discussion:**

Through multi-dimensional data audits collected by clergy and congregants, the *Our Voice* method fostered meaningful local data collection and analysis to identify and address community factors that may impact health. Findings provide evidence that action plans developed and implemented by faith-based community members may have a broad reach beyond the congregation and create potential opportunities for co-developing community-based health promotion programming.

## 1 Introduction

In the United States (US), nearly 70% of individuals identify with a religious tradition, most of which represent the Christian faith, with ~33% attending religious services monthly (1). Faith-based organizations (i.e., churches), with their broad reach, have proven effective as convenient settings for multi-level chronic disease prevention and health promotion programming (2–6). Previous faith-based health promotion programs targeting the social and built environments both within (e.g., church policies, messaging) and around the church (e.g., nearby park use) have found positive effects in both individual- and community-level health outcomes (e.g., physical activity behavior, diet, weight loss, social cohesion, and/or faith leader buy-in [hereafter *clergy*]) (3, 4, 7, 8).

Clergy are trusted gatekeepers and public health messengers (9, 10) and their involvement, support, and promotion of health initiatives can be instrumental to successful implementation of health promotion programming (11). However, many clergy report individual, social, and structural barriers (e.g., 24 h/day, 7 days/week demands on their time) hindering their own ability to engage in health behaviors (12, 13), potentially contributing to the high prevalence of chronic physical and mental health conditions (e.g., obesity, depression) seen among the clergy population (12, 14, 15). Despite recent literature documenting how multi-level, congregation-focused health promotion may also have potential positive downstream effects on clergy (e.g., increasing physical activity engagement) (14), few studies have jointly addressed the health needs of both clergy and congregants. In fact, research with clergy and other church leaders show that their health needs and priorities might differ than that of the congregants they serve (16). To date, there is limited research on how participatory action approaches and processes (e.g., citizen science) could involve both clergy and congregants in identifying and co-creating strategies that support the health and well-being of their congregation.

Globally, citizen science has become a popular approach to enhance chronic disease prevention efforts (17, 18). Across the literature, however, citizen science research varies in the level of community member involvement that occurs, from “for the people” forms of citizen science (e.g., community members provide information to researchers, after which their scientific participation typically ends); “with the people” citizen science (e.g., community members participate in systematic forms of data collection, with the data then typically pushed to scientists); and “by the people” citizen science (e.g., participation across the fuller range of research processes and activities) (19, 20). While previous research documents citizen science approaches across a diverse range of scientific disciplines (18), the Stanford *Our Voice* method, informed by community-based participatory research principles and the Socio-Ecological Framework, has emerged as a feasible yet effective approach due to its adaptability across contexts and populations as well as the intentional focus on contextually relevant solutions generated “by the people” (21–23).

The evidence-based *Our Voice* method consists of 4-steps: Discover, Discuss, Activate and Change (21). In the Discovery phase, community members are invited to collaborate in developing a guiding question for taking photos and recorded narratives, and then uses the *Our Voice* Discovery Tool mobile app (24) to collect data related to that question. Through the Discovery Tool, citizen scientists can document their lived experience data from their local contexts (e.g., geotagged photos, audio/text narratives, simple sentiment ratings based on emojis) (24). The citizen scientists then gather to discuss their data, build consensus around common themes, and brainstorm (Discuss). They then learn how to partner with local decision-makers to activate feasible solutions and action steps (Activate) to address shared priorities for community-level change (Change) (21).

*Our Voice* has been applied globally to address health-related inequities and increase opportunities for healthy living, including physical activity promotion (19), enhancing safe routes to school (25), identifying factors impeding healthy aging (23), and increasing access to healthy foods (21). While *Our Voice* is effective in bringing about positive changes to social and built environments across the globe, no researchers, to date, have adapted and tested this method within churches or other faith-based organizations. Such settings have been deemed crucial for addressing community-level health needs and reducing health disparities (26). Thus, the purpose of this paper is to: describe a pilot study adapting and implementing *Our Voice* within faith-based settings guided by the Reach Effectiveness Adoption Implementation Maintenance (RE-AIM) framework to maximize reach, feasibility, and potential for sustainability (27–29). We will report strategies used to seek input from clergy, congregants, and other trusted community members to enhance reach and ensure *Our Voice* content aligned with cultural traditions; and evaluate its potential for identifying contextually relevant factors and co-creating opportunities for the congregation to support both clergy and congregant health and well-being.

## 2 Materials and methods

### 2.1 Study setting

Alaska, with its population of 737,270, is ranked in the top 15 states for cultural diversity (30) with residents identifying as White (62%); American Indian/Alaska Native (16%), Hispanic (8%), Asian (7%), Black or African American (3%) and Native Hawaiian/Pacific Islander (2%) (31). While approximately 260,000 (~35%) Alaskans are involved with a religious congregation (32), most of who identify with the Christian tradition, no study to date has documented efforts to engage Alaskan clergy and their congregations in health promotion and chronic disease prevention research.

### 2.2 Procedures

#### 2.2.1 Pre-implementation phase: adapting our voice to faith-based settings

Using RE-AIM as a guide (27), for approximately 4 months prior to study implementation, study protocol and materials were adapted through an iterative process informed by community and academic expertise, with the goal of diversifying the reach and representativeness of participating communities, and the alignment of content and procedures with churches, as the “adopting” and “implementing” settings. RE-AIM use in implementation planning assured that the research team paid attention to key domains associated with successful implementation (27–29). Specifically, the research team incorporated feedback from community members and academic experts and discussed who will benefit from the approach and strategies selected, and who might be missed (*Reach*); what desired impact will selected strategies produce and how will they measure it (*Effectiveness*); what characteristics of each setting (including features of the space, built/social environment, and people responsible for implementing the strategies) are relevant to implementation decisions (*Adoption*); what evidence, barriers, facilitators, and costs contributed to strategy selection, and what adaptations were made to improve fit and feasibility (*Implementation*); and how will the adopting setting maintain strategies and focus on health promotion beyond the study (*Maintenance*) (27–29). See Table 1 for protocol and *Our Voice* terminology adaptations inspired by community feedback and Figure 1 for an overview of study processes from pre-implementation to implementation of the *Our Voice* method within faith-based settings. This study was approved by the University of Alaska-Anchorage’s Institutional Review Board.

**Table 1:** Summary of adaptations to *Our Voice* protocol to enhance relevance to faith-based settings.

**Domain**	**Original protocol**	**Adapted protocol (Co-design)**
*Our Voice* language and guiding question	“Citizen Scientist” “How does your church support or promote healthy eating and physical activity?”	“Lay-Scientist” “What features within and surrounding your church reinforce or do not reinforce your physical, nutritional, mental, or spiritual wellbeing?”
Faith-based connection on study materials	N/A	Scripture tells us that our physical, nutritional, spiritual, and mental wellbeing are important for our own health and the health of our community. (1 Timothy 4:8; Genesis 1:29; 1 Corinthians 10:31; Mark 12:30–31; Philippians 4:6–7) Scripture also tells us that we do not have to do it alone. To help each other improve and encourage and serve one another is central to our spiritual beliefs. (Matthew 18:20; Proverb 27:17; Philippians 2:3–4)
Incentives	$20 Pre-Post Gift Cards	$20 Pre-Post Gift Cards (Individual Level) $800 implementation contract as seed funding (Church Level)
Community partnership Agreement	N/A	Memorandum of Understanding signed by each participating church

**Figure 1:**
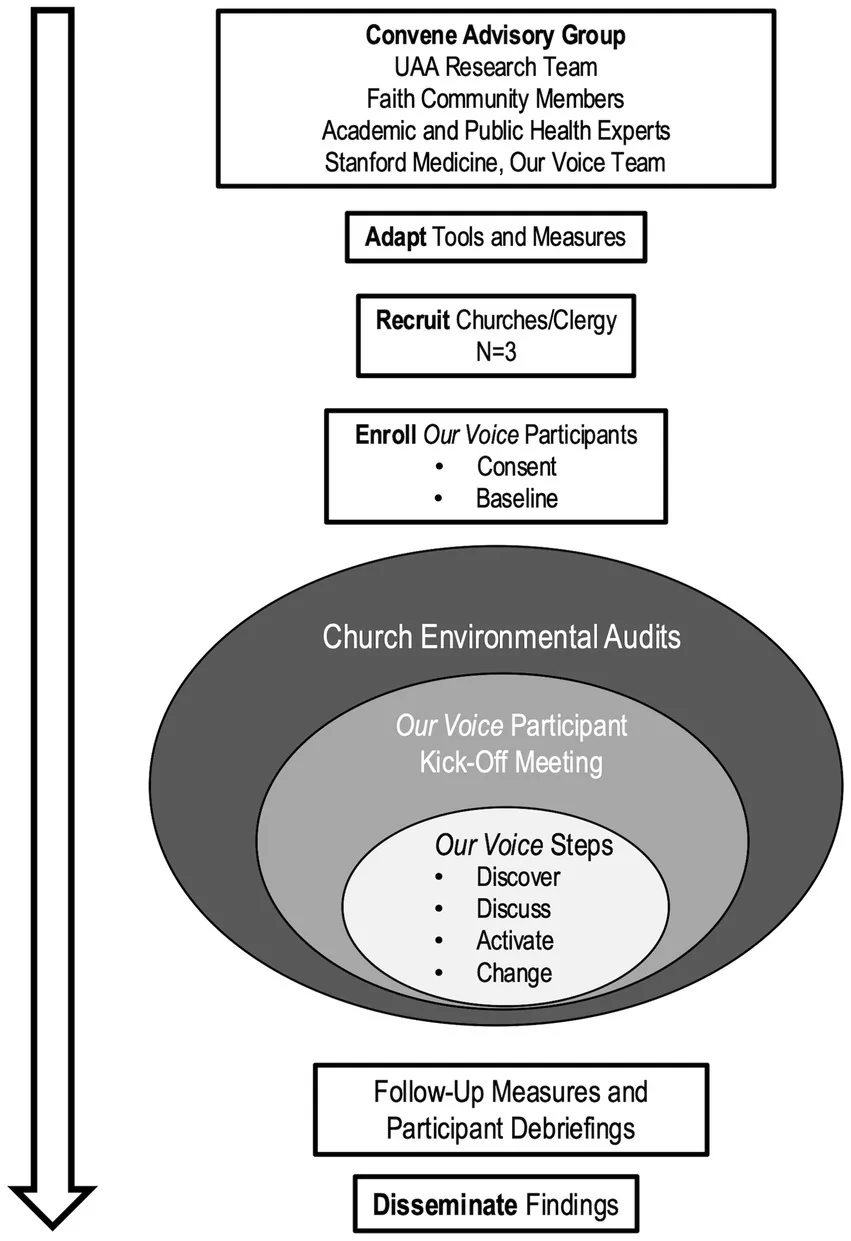
Schematic highlighting study processes from pre-implementation to implementation.

##### 2.2.1.1 Project outreach (months 1–2)

In this planning phase, research staff shared the original study design and protocol with clergy affiliated with faith community networks as well as local lay-leaders with a history of engaging faith-based organizations in addressing public health priorities. Feedback provided from a community stakeholder prompted research staff to reevaluate the benefits and implementation of this study, particularly regarding church capacity to implement action plans, from a cost and personnel perspective. Based on this feedback, additional funds were obtained from the Alaska Department of Health to provide $800 stipends for participating churches, to be used as seed funding for implementing at least one priority action plan activity developed through the *Our Voice* process.

##### 2.2.1.2 Community-academic expert advisory panel (month 3)

In the next phase, research staff invited established community and academic partners to a structured, three-hour “project kickoff” meeting. The multidisciplinary advisory panel (*N* = 6) represented diversity across race/ethnicity, age, faith tradition, and academic disciplines (e.g., clergy, congregants, public health experts). The panel reviewed and provided feedback on study design, materials, and methods as well as standardized *Our Voice* terminology and data collection. Based on feedback from the panel, instead of citizen scientists, one recommendation was to refer to participating clergy and congregants as “lay-scientists” to align with cultural tradition in faith-based settings.

##### 2.2.1.3 Stanford our voice technical training and establishing community expectations (months 3–4)

In the third phase, research staff, in collaboration with the *Our Voice* technical team, incorporated community-inspired feedback and customized the project’s specific guiding question to be used for Discovery Tool data collection. Additionally, project research staff attended two in-depth virtual trainings on the *Our Voice* method (Discovery, Discuss, Activate, Change) including how to effectively engage community members to use the Discovery Tool to collect meaningful lived experience local environment data, and how to facilitate productive follow-up discussions to identify and prioritize themes and create action plans (24). An *Our Voice* instruction handout, presented to lay-scientists at the Discovery Meeting, was also developed that included faith-based messaging and Discovery Tool training (see Figure 2).

**Figure 2:**
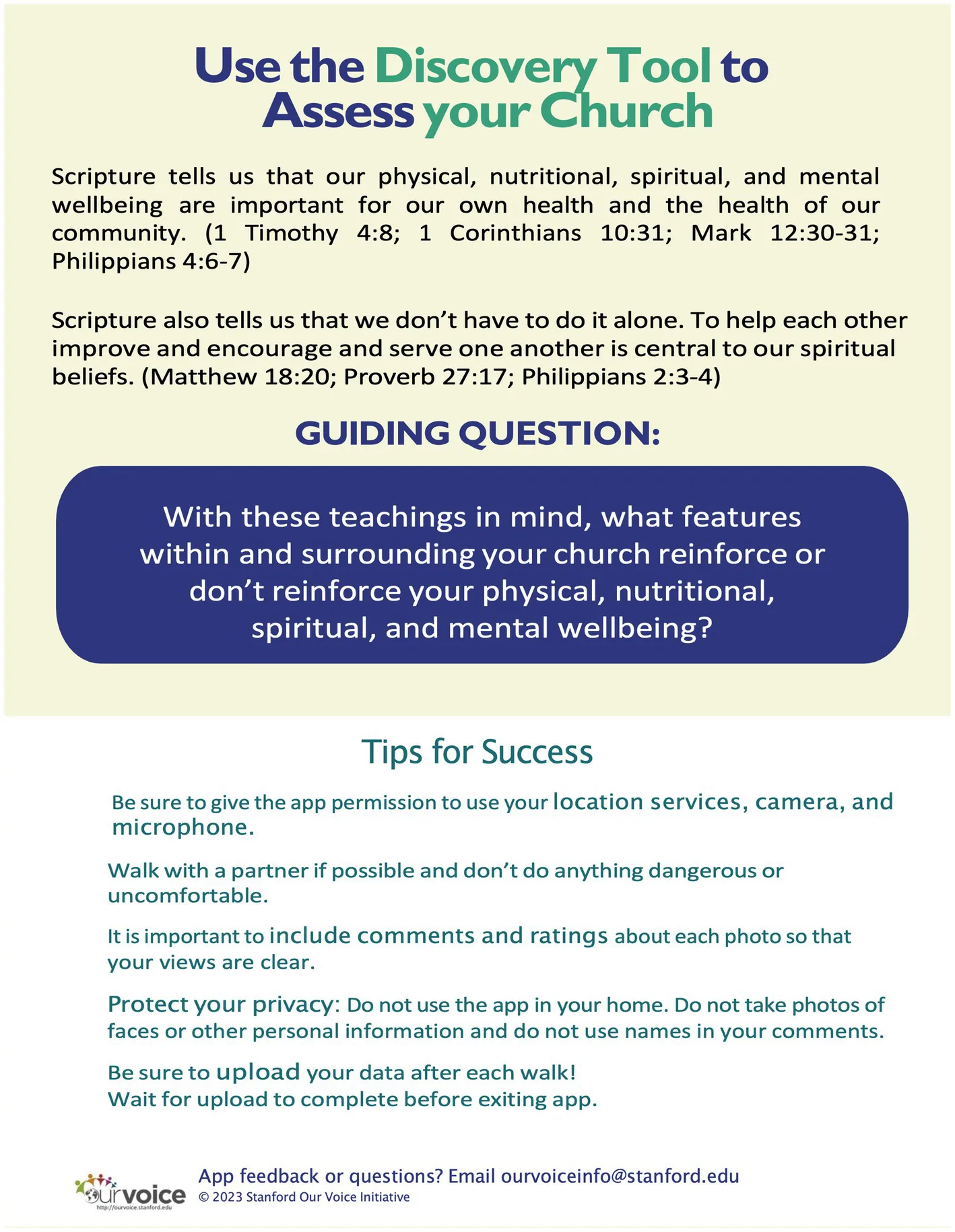
Example packet item created in collaboration with the *Our Voice* expert technical team using messaging inspired by community feedback. Created using the Our Voice framework (https://ourvoice.stanford.edu).

To establish concrete expectations for participating community members, project research staff designed a Memorandum of Understanding (MOU) that outlined the study purpose and protocol for all partners (research staff, church leaders, lay-scientists). The MOU was developed to be signed by the lead researcher and a point of contact at each church and could be revoked by the church at any point during the study.

##### 2.2.1.4 Community finalization (month 4)

In the fourth and final step of project planning, a trusted and representative clergy member with longstanding community and institutional knowledge reviewed and approved the near finalized version of the revised protocol and materials in a face-to-face meeting.

#### 2.2.2 Implementation phase: piloting our voice within faith-based settings

##### 2.2.2.1 Recruitment and project rollout

Across a year (2024–2025), a staggered approach was employed to test the *Our Voice* method with one participating church at a time. Through extensive community partner networks and direct outreach from research staff (e.g., email, one-on-one meetings, community presentations), clergy were purposively recruited from three Christian churches in Alaska representing diverse community characteristics (e.g., race/ethnicity, age, gender, church size, location) to pilot the *Our Voice* method (21–23).

To reach representative congregants, we partnered with clergy from each church setting to use community-aligned recruitment methods. The recruitment strategies varied across church settings, as follows: (Setting #1) Lay-scientists were hand-selected, a liaison was appointed to work directly with research staff to coordinate data collection and community meetings; (Setting #2) Research staff were invited to present the project information during a worship service and connected to representative congregants via email; and (Setting #3) Project information was shared from the pulpit and then research staff was connected to interested congregants via email. Of note, before any data collection activities, all lay-scientists provided informed consent and research staff worked with a church point of contact to explain, review and sign the MOU.

##### 2.2.2.2 Researcher-led environmental audit

Prior to participant-led data collection activities with each participating church, research staff, in coordination with an established point of contact (e.g., clergy, church staff), completed an environmental audit using a standardized environmental assessment tool to describe and document the observed built environment features of each church setting (see measures and data collection).

##### 2.2.2.3 Our voice implementation across church settings

Processes and lessons learned were documented (i.e., what worked, what did not work) and applied with the next participating church. Two church settings were in driving distance from the university setting; thus, each data collection and community meeting followed our planned implementation flow, with 14–21 days between each face-to-face meeting (Discovery [Data Collection Meeting], Discuss [Community Meeting #1], Activate [Community Meeting #2]). A third church was in a rural setting that is not on the road system and, can only be reached by boat or plane. At this setting, the co-pastor and research staff co-designed a condensed approach where the lead researcher would be housed at the church setting and facilitate all *Our Voice* meetings in person within a 4-day period, with the goal of maximizing lay scientist engagement within the constraints of project funding and timelines. Based on community notes and process documentation, we describe below the adaptation and implementation of the *Our Voice* method (Discover, Discuss, Activate) across church settings.

###### 2.2.2.3.1 Discover

In the Discovery meeting (data collection meeting) with church setting #1, research staff presented each lay-scientist (clergy and congregants) with a packet detailing the study purpose, the connection between the current study and the Bible, the purpose of *Our Voice* and a brief overview of the Discovery Tool. Research staff then distributed project mobile phones (i.e., Tracfone®) to each lay-scientist, provided a brief training on the Discovery Tool (~5–10 min) and then encouraged lay-scientists to walk in groups of 3 or 4 to take photos and record narratives of features within and surrounding the church that did or did not reinforce their physical, nutritional, mental and spiritual well-being (~30–40 min). Once lay-scientists became comfortable with the Discovery Tool, many walked individually around the church setting to collect data. Churches #2 and #3 followed a similar protocol, but lay-scientists were given the option to walk individually or in groups (depending on group size).

After the Discovery meeting, research staff analyzed all photos and narratives guided by the health domains presented in the guiding question as *a-priori* themes (physical, nutritional, mental, spiritual) to better understand the important church features lay-scientists were documenting. In addition to the *a-priori* themes, and based on community notes and narratives recorded, community wellbeing was added as an important theme within the discussion meeting.

###### 2.2.2.3.2 Discuss

In the Discussion meeting (community meeting #1) at church setting #1, lay-scientists were given an individualized packet containing their photo and narrative data collected with the Discovery Tool. Given the number of lay-scientists and the size of the meeting room, research staff tested the process of pre-selecting groups of 2 to 3 lay-scientists with a similar number of photos taken and asked them to discuss as a group the meaning behind each photo and narrative. Next, with flip charts representing *a priori* themes (e.g., physical, nutritional, mental, spiritual, community well-being) set up around the room, each lay-scientist sorted their photos based on the themes and placed their photos by the relevant flip chart. Participants then used sticky note pads to write their perceptions of church “strengths” and “opportunities for growth” corresponding to each theme, and posted them on the flip chart. Research staff and lay-scientists then worked together to identify and develop priority areas for action. Based on lay-scientist engagement from church setting #1, this method was applied across all church settings. Of note, due to the group size and meeting room size of church setting #2 and #3, lay-scientists were not split into pre-selected groups to discuss photos, rather each lay-scientist had the opportunity to share with the entire group the meaning behind each photo and narrative.

###### 2.2.2.3.3 Activate

In the Activation meeting (community meeting #2), beginning with church setting #1, research staff and lay-scientists synthesized data collected across all meetings and developed contextually-relevant action plans. After the activation meeting, a document detailing each plan and next steps were prepared by research staff, disseminated to each church, and validated by group members. Once each confirmed their action plan(s), research staff worked with the university budget office and the church point of contact to distribute the $800 implementation seed funds.

### 2.3 Measures and data collection

#### 2.3.1 Pre-post survey

At baseline, clergy and congregants provided information on their age, gender, race/ethnicity, education, and denomination as well as their physical activity engagement using the Godin-Leisure Time Exercise Questionnaire (Active, Insufficiently Active) (33). At baseline and follow-up, clergy and congregants were asked to rate how confident they were that they could stick with physical activity or healthy eating on an 11-point scale (0–100%) using the 4-item Physical Activity (PASE; Example item: “How confident are you that you can be physically active even if you if have to try several times until it works”) and 4-item Healthy Eating Self-Efficacy questionnaires (HESE; Example item: “How confident are you that you can stick with eating healthful foods even if you need a long time to develop necessary routines”) (34). Language in one HESE item was adapted for the purpose of this study (“… even if you have to rethink your entire way of nutrition” to “even if you have to rethink your entire way of healthy eating”). Lay-scientists were also asked to rate their perceptions of social cohesion and agency on a 5-point scale (Strongly Disagree to Strongly Agree) using a culturally adapted version of the 6-item *Our Voice*-employed Social Cohesion and Agency scale (Example item: “This is a church where people support each other”) (35). Of note, one item (“My pastor should have time for their own self-care”) was added to capture clergy and congregant perceptions towards clergy health.

PASE and HESE questionnaire items were summed, and an average score was calculated to create a baseline and follow-up score (34). Social Cohesion and Agency scale items were numerically coded (e.g., Strongly Disagree = 1; Strongly Agree = 5) and compared across baseline and follow-up. Lay-scientists were asked to complete surveys evaluating their experiences at baseline (before the Discovery meeting) and at follow-up (after the second group meeting) through an online survey platform (i.e., Qualtrics). Lay-scientists received a $20 gift card for the completion of each survey ($40 total).

#### 2.3.2 Church environmental audit tool

Research staff initially assessed the health promotion environment of each church using a validated church environmental audit tool (ChEAT) (36). After reviewing project materials shared by participating clergy at their church, a lay-scientist expressed concern to research staff about the negative connotation of the ChEAT acronym, particularly in faith-based settings (*hereafter* EAT). The EAT included a checklist of items related to physical activity (e.g., space, equipment) and healthy eating resources (e.g., kitchen, vending machines) that were present (yes, no), usable (yes, no), and/or in good condition (yes, no) within and around the church. For the purposes of this study, an open text item was added to the checklist to provide contextual information on the eating environment (e.g., fast-food restaurants, grocery stores) within walking distance from the church (“Describe the healthy eating environment around the church.”). At each church, one to two trained research staff members completed the EAT as they walked through the facility with an established point of contact (e.g., clergy, church staff). After each church walkthrough, research staff then discussed independent observations to identify and build consensus for any initially discrepant findings.

#### 2.3.3 Discovery tool

Lay-scientists were trained on using the Discovery Tool, a multi-lingual smartphone app, which was downloaded from either the Apple or Google app stores on designated project mobile phones. The Discovery Tool records geo-tagged photos, narratives (e.g., audio, text) and their sentiment ratings (as positive, negative, or of mixed impacts for community health) along with route maps to document the features within and surrounding their church that reinforced or did not reinforce their physical, nutritional, mental, and spiritual well-being (24).

### 2.4 Data analysis

Descriptive and frequency analyses were conducted for all church- and individual-level characteristics (e.g., age, gender, race/ethnicity, church size) using SPSS v.26 (IBM Corp, 2021). Using deductive coding, a qualitative synthesis of EAT checklist items (e.g., indoor physical activity and healthy eating opportunities; outdoor physical activity and healthy eating opportunities) was conducted to summarize the social and built environment of each church setting, particularly if items were present (yes, no), usable (yes, no) and in good condition (yes, no). Community notes of observations by research staff and additional information given by the church point of contact (e.g., clergy, church staff) were used to provide additional detail to health promotion opportunities within and around the church.

Using the Stanford University project-specific and data-protected *Our Voice* secure data portal, Discovery Tool data (e.g., photos, audio/text narratives, sentiment ratings accompanying photos + narratives) were qualitatively examined to produce the total count as well as common themes within and across church settings. An audit trail of meeting notes (data collection meetings, community meetings, staff meetings) was used to document lay-scientist engagement in the *Our Voice* process (e.g., meeting attendance, ideas generated, action plans developed).

Due to the reasonably small sample size and non-normal data, Wilcoxon signed ranked tests (sample > 10) were conducted to assess differences across pre-post outcomes (PASE and HESE; Social Cohesion and Agency). Cases with missing follow-up data (*n* = 3) were automatically excluded from the analysis. Significance level for all analyses was set at 0.05.

## 3 Results

### 3.1 Church setting characteristics and descriptions

See Table 2 for descriptions of the three participating churches. Church settings varied in size (<100 to 101–250 members), denominational affiliation (Baptist, Nazarene, United Methodist), and geographic location (two urban, one rural). Qualitative synthesis of EAT findings revealed the presence of a wide variety of opportunities for health promotion within or proximal to the church (e.g., basketball gyms, local parks, spacious sanctuaries, grocery stores, restaurants, community gardens, kitchen/cooking stations). These features were then discussed during *Our Voice* meetings to further understand their potential to support identified priorities, as well as any barriers to their use.

**Table 2:** Descriptions and characteristics of the three Alaskan church settings where the *Our Voice* method was implemented.

Setting	Church size	Denomination	Location
Church #1	101–250	Baptist	Urban
Church #2	<100	United Methodist	Suburban
Church #3	≤10	Church of the Nazarene	Rural

Environmental audit tool findings: qualitative synthesis	
Church #1	*Indoor PA Opportunities:* Spacious Fellowship Hall; Full-Court Basketball Gymnasium; Multiple Stairwells; Exercise Equipment (e.g., rubber bands, yoga mats, jump ropes, videos, basketballs). All items were usable and in good condition. *Outdoor PA Opportunities:* Walking Path and Green/Open Space (adjacent to the facility); Spacious Paved Parking Lot; Portable Basketball Goals. All items were usable and in good condition. *Indoor HE Opportunities:* Commercial Kitchen (e.g., large industrial cooking stations and appliances). All items were usable and in good condition. *Outdoor HE Opportunities:* Outdoor Grill (usable and in good condition). No eating establishments or grocery stores proximal to church facility. *Media Assessment:* TV Screens Near Fellowship Hall; Bulletin Boards throughout Facility. All items were usable and in good condition, but no health promotion materials were present.
Church #2	*Indoor PA Opportunities:* Spacious Fellowship Hall; Stairwell. All items were usable and in good condition. *Outdoor PA Opportunities:* Spacious Paved Parking Lot; Open Space; Playground/Play Area (e.g., fixed play equipment). All items were usable and in good condition. While not proximal to church, three parks/walking trails located nearby (≤1.5 miles). *Indoor HE Opportunities:* Standard Kitchen (e.g., refrigerator, freezer, oven, and other appliances similar to home kitchen). All items were usable and in good condition. *Outdoor HE Opportunities:* Vegetable Garden (usable and in good condition). No eating establishments or grocery stores proximal to church facility. *Media Assessment:* One Bulletin Board in Center of Facility. Item usable and in good condition, but no health promotion materials were present (except for church vegetable garden advertisement).
Church #3	*Indoor PA Opportunities:* Spacious Fellowship Hall; Half-Basketball Gymnasium; Stairwell; Exercise Equipment (e.g., basketballs). All items were usable and in good condition. *Outdoor PA Opportunities:* N/A *Indoor HE Opportunities:* Standard Kitchen Appliances (e.g., refrigerator, countertop similar to home kitchen); Commercial Stove/Oven. All items were usable and in good condition. *Outdoor HE Opportunities:* Eating establishments and grocery store proximal to church facility (≤1.0 Miles). *Media Assessment:* One Bulletin Board in Center of Facility. Item usable and in good condition, but no health promotion materials were present

PA = Physical Activity; HE = Healthy Eating; Indoor PA/HE Opportunities = Items or Features Available within Church Facility; Outdoor PA/HE Opportunities = Item or Features Available Around or Proximal to Church Facility.

### 3.2 Lay-scientist demographics

See Table 3 for demographic characteristics of lay-scientists across each church setting. Lay-scientists (*N* = 17) across three churches completed the baseline survey. On average, lay-scientists were 57.3 years of age (SD = 11.4), had a college degree (*n* = 14), were mostly physically active (*n* = 9), church members (*n* = 9), female (*n* = 10) and self-identified as Black or African American (*n* = 6), White (*n* = 10), or American Indian or Alaska Native (*n* = 1).

**Table 3:** Demographic characteristics of lay-scientists who participated in an *Our Voice* study within faith-based settings.

Variable	Total (*N* = 17)	Church #1 (*n* = 7)	Church #2 (*n* = 6)	Church #3 (*n* = 4)
	*n*	*n*	*n*	*n*
Age, Mean (SD)	57.3 (11.4)	60.3 (7.1)	50.2 (16.0)	66 (62.8)
Race/Ethnicity				
Black or African American	6	6	----	----
White	10	1	6	3
American Indian or Alaska Native	1	----	----	1
Gender				
Male	6	3	2	1
Female	10	4	3	3
Non-Binary	1	----	1	----
Education				
High School or Less	1	----		1
Some College/Two-Year Degree	2	1	1	----
College or Higher	14	6	5	3
Physical activity status				
Active	7	4	3	0
Insufficiently Active	10	3	3	4
Church role				
Clergy/Pastor	4	1	1	2
Church Staff	3	2	1	----
Church Member	9	3	4	2
Other	1	1	----	----

### 3.3 Our voice implementation

Through three meetings (data collection as well as two community meetings), lay-scientists were engaged in systematically collecting and analyzing lived experience data detailing the health promotion environment within and surrounding their church (Discover, Discuss, Activate). Lay-scientists then worked together to implement action plans to address priorities identified in the community (Change [Action Plans and Short-Term Indicators]). See Table 4 for the full description of *Our Voice* implementation across church settings (e.g., engagement, meeting attendance, exemplary narratives).

**Table 4:** Description of the *Our Voice* implementation process across three Alaskan church settings.

Variable	Church #1	Church #2	Church #3
**Our voice implementation**	Discovery, Discussion, and Activation meetings held ~2 weeks apart (Total project duration = ~6 Weeks)	Discovery, Discussion, and Activation meetings held ~2 weeks apart (Total project duration = ~5 Weeks)	Discovery, Discussion, and Activation meetings held ~1 day apart (Total project duration = ~4 Days)
Project meeting dates			
*Discovery Phase*	July 18th, 2024	February 12th, 2025	April 13th, 2025
*Discussion Phase*	August 8th, 2024	February 26th, 2025	April 15th, 2025
*Activation Phase*	August 22nd, 2024	March 12th, 2025	April 16th, 2025

**Discovery phase (data collection meeting)**			
Discovery walks	7 Walks	6 Walks	4 Walks
Photos taken	71 Photos	67 Photos	78 Photos
Sentiment ratings (total)^a^	60 Total Ratings	57 Total Ratings	57 Total Ratings
*Positive Rating*	53 Positive	49 Positive	36 Positive
*Negative Rating*	1 Negative	6 Negative	7 Negative
*Mixed Rating*	6 Mixed	2 Mixed	14 Mixed
Narratives recorded (total)	66 Narratives	65 Narratives	74 Narratives
*Audio*	50 Audio	39 Audio	55 Audio
*Text*	16 Text	26 Text	19 Text
Exemplary narratives	*“I took a picture of the blank boards because when we talk about health, particularly as it relates to eating, not many, if any, of our boards have anything on it that speaks to healthy eating, physical activity, and things of that nature.”*	*“…We have been given some things from [name blinded], which is right next door, so that's a relationship with the church and the elementary school. They have given us gardening supplies before so that's an area where we could probably kind of work to promote gardening and healthy eating.”*	*“...this picture of the [name blinded] center fellowship hall, because it represents a great space to be able to do things with the community. We have the unhoused service center [name blinded] here in the evenings, but what about health clinics quarterly or some other kind of community outreach that draws faith and health together?”*

**Discussion phase (community meeting #1)**			
Community meeting attendance			
*Community Meeting #2*	6/7 Attendees	5/6 Attendees	4/4 Attendees
Opportunities for growth (total count)	25 Ideas	18 Ideas	28 Ideas
*Physical wellbeing*	6 Ideas	4 Ideas	5 Ideas
*Nutritional wellbeing*	4 Ideas	5 Ideas	5 Ideas
*Mental wellbeing*	6 Ideas	2 Ideas	6 Ideas
*Community wellbeing*	5 Ideas	5 Ideas	7 Ideas
*Spiritual wellbeing*	4 Ideas	2 Ideas	5 Ideas
Opportunities for growth (exemplary quotes)	“*More community organizations/persons using the facility” (Community)* *“Promote more physical activity through programs offered in the city” (Physical)* *“Use nutritional professionals/persons in congregation to speak on nutrition” (Nutritional)*	*“Garden – involving [local] school with [church] garden…” (Community)* *“Opportunities to plan activities in nature” (Community)* *“Make the play area more open/available to neighbors” (Physical)*	*“Become trauma-informed faith community” (Mental)* *“Facebook posts to encourage spiritual health” (Spiritual)* *“Food and cooking support for unhoused service center [name blinded] (Nutritional)* *“Offer mental health wellness class” (Mental)*

**Activation phase (community meeting #2)**			
Community meeting attendance			
*Community Meeting #2*	5/7^b^ Attendees	5/6^c^ Attendees	4/4 Attendees
Action plans			
*Priority 1*	Implemented	Implemented	Not Yet Implemented
*Priority 2*	Implemented	In-Process	----
*Priority 3*	Implemented	Not Yet Implemented	----
*Priority 3a*	Not Yet Implemented	----	----

^a^ Some photos missing sentiment ratings; ^b^Two lay-scientists did not attend due to health reasons; ^c^One lay-scientist did not attend due to health reasons.

#### 3.3.1 Discover

Across the three church settings, lay-scientists completed 17 discovery walks, using the Discovery Tool mobile app to take 216 photos and record 205 narratives (144 recordings, 61 text) about the features of their church that reinforced or did not reinforce their physical, nutritional, mental, and spiritual well-being. Overall, the majority of church features within and surrounding the church setting had positive sentiment ratings (n = 138). Examples of features rated less favorably included accessibility (e.g., poor lighting, architectural barriers for congregants who are older or with disabilities), cleanliness of the church environment (e.g., cluttered rooms), older infrastructure, and sidewalks on church property not routinely kept clear during winter months. See Figures 3–5 for example photos, narratives, and sentiment ratings collected using the Discovery Tool across church settings.

**Figure 3:**
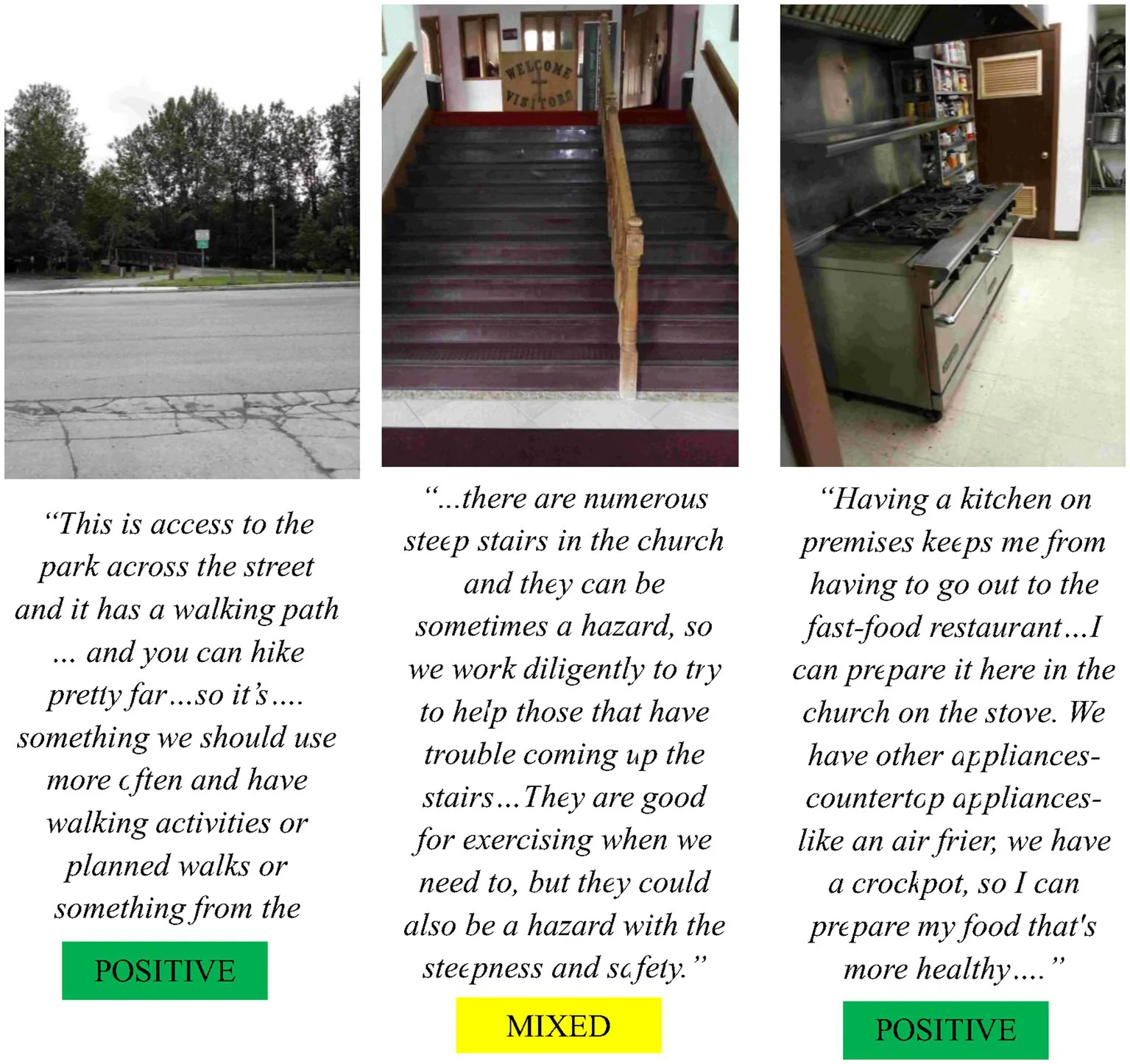
Exemplary Discovery Tool photos and narratives with accompanied sentiment ratings from church #1.

**Figure 4:**
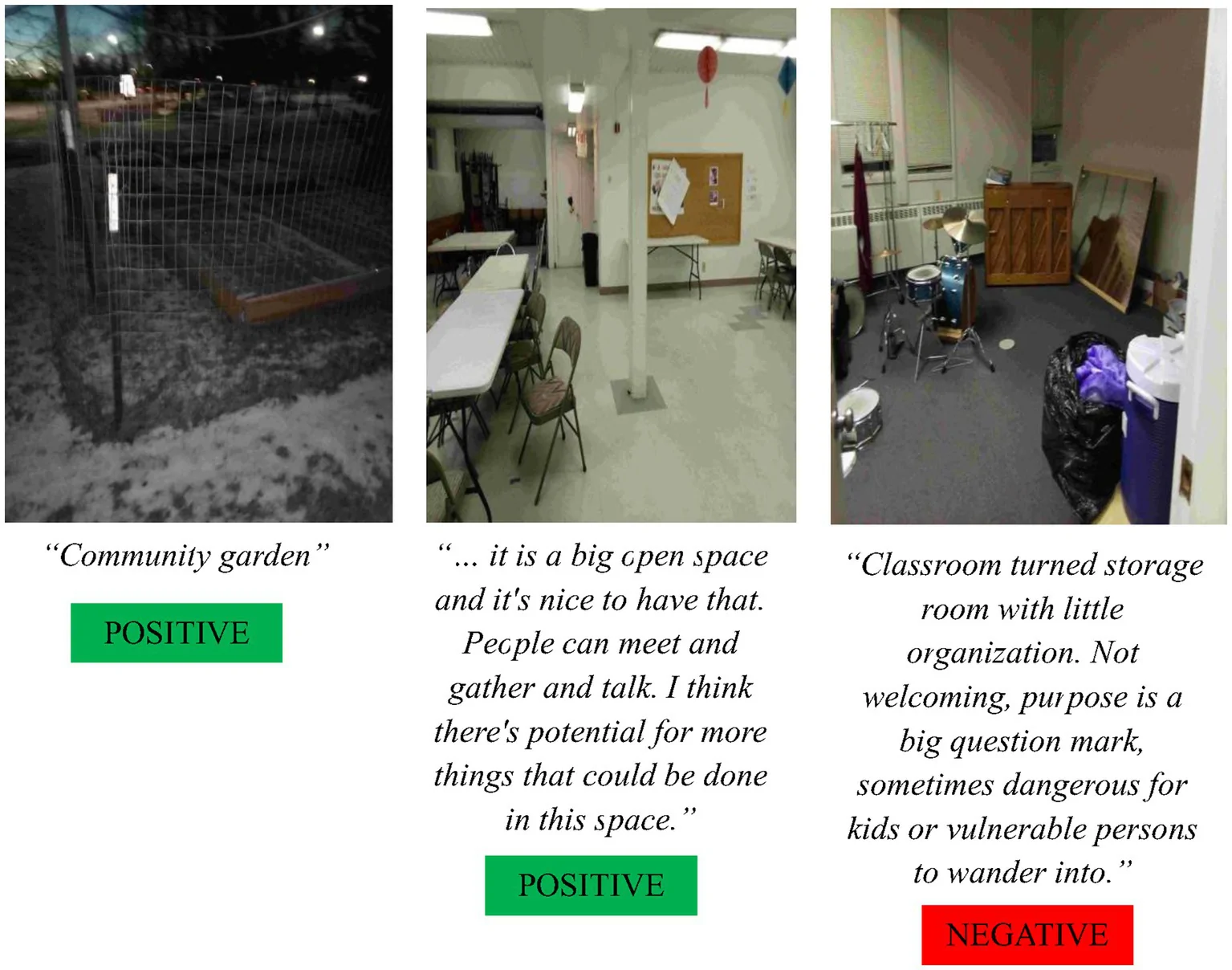
Exemplary Discovery Tool photos and narratives with accompanied sentiment ratings from Church #2.

**Figure 5:**
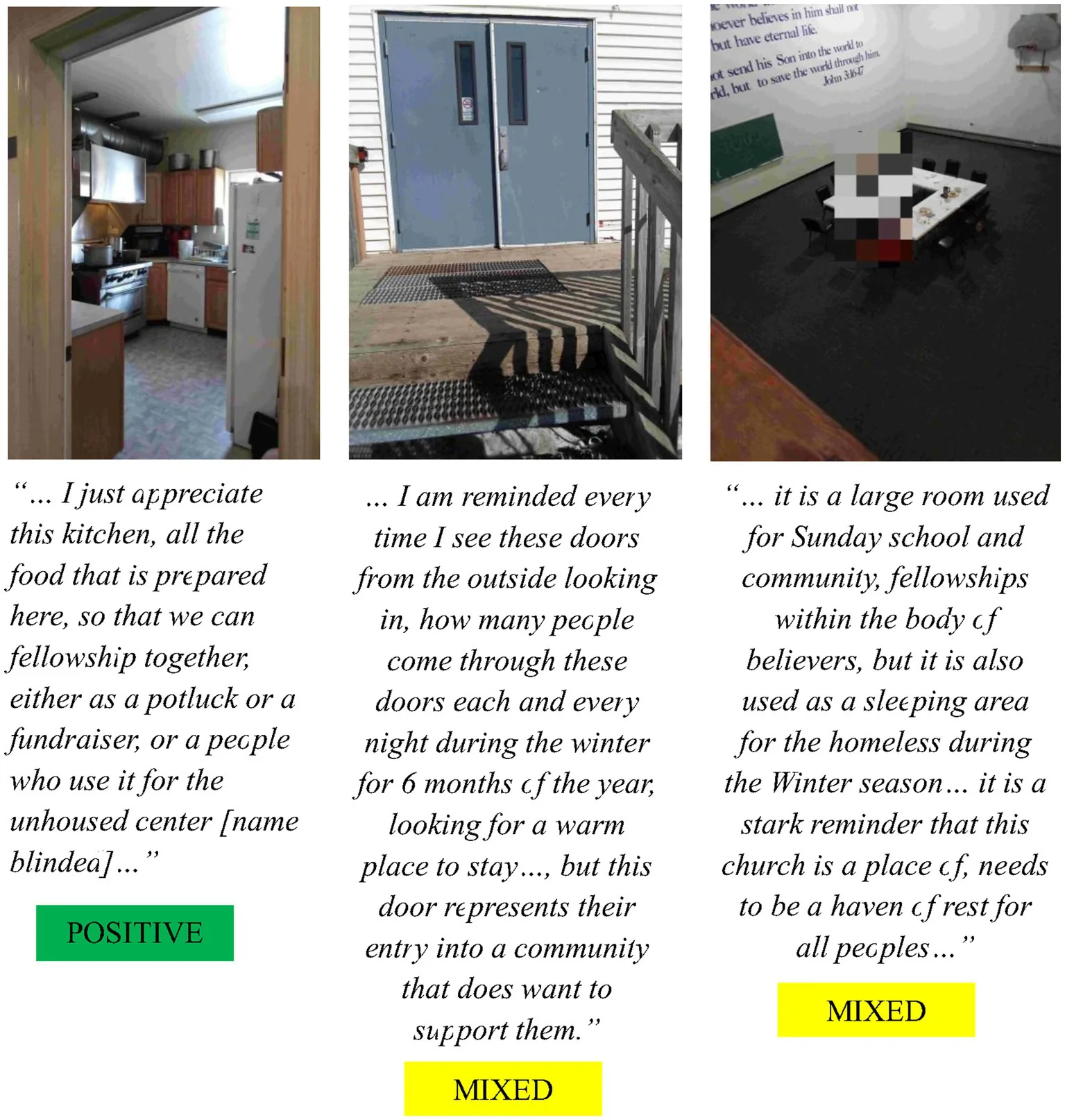
Exemplary Discovery Tool photos and narratives with accompanied sentiment ratings from church #3.

#### 3.3.2 Discuss

Across each setting, lay-scientists identified 54 opportunities for growth (i.e., idea generation) regarding community (*n* = 17), physical (*n* = 15), mental (*n* = 14), nutritional (*n* = 14), and spiritual wellbeing (*n* = 11) at their respective churches. See Figure 6 for depiction of the process lay-scientists and research staff utilized to discuss opportunities for growth across church settings.

**Figure 6:**
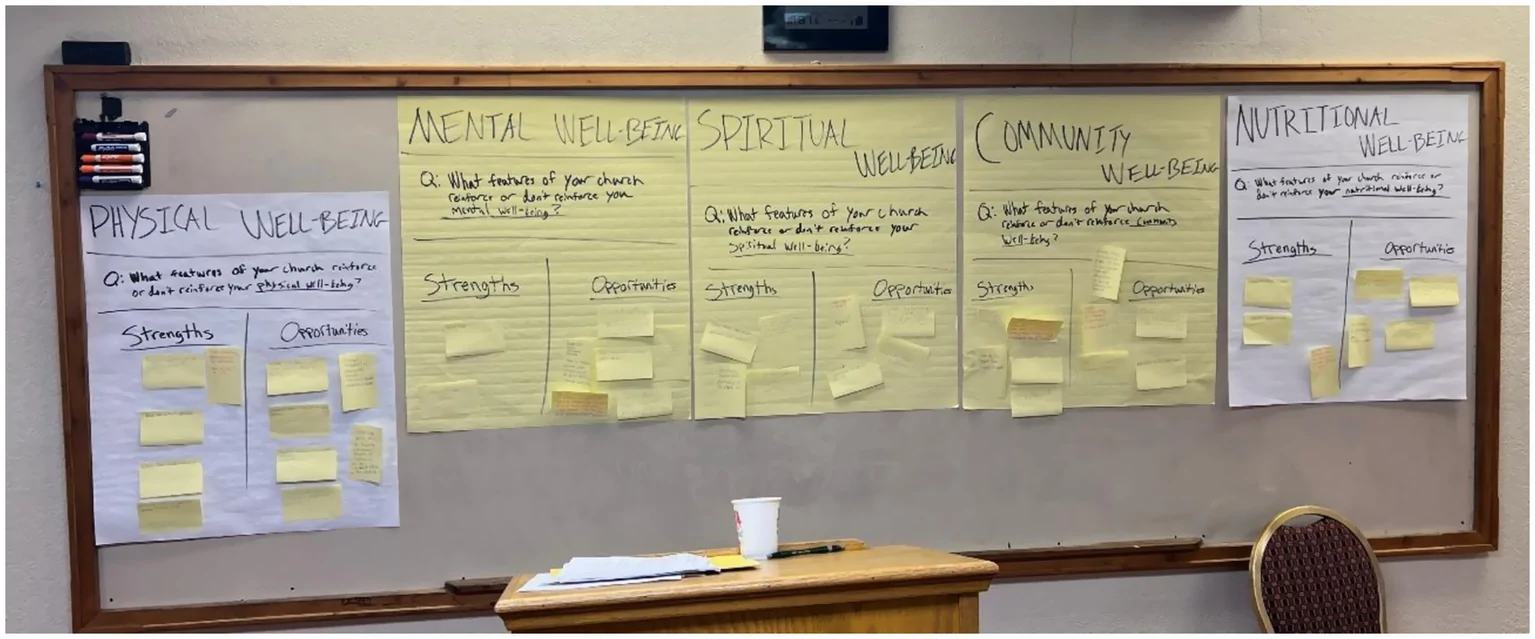
Photo depicting the process used to synthesize opportunities for growth in the Discuss Phase.

#### 3.3.3 Activate

At church settings #1 and #2, research staff and lay-scientists synthesized data collected across all meetings to develop three to five specific action plans. Due to the size of church setting #3, the variation in ideas generated, differences in priorities across congregants and clergy, and extraneous community factors impacting action plan development (e.g., lack of church volunteers, organization for people experiencing homelessness occupying church gymnasium), research staff encouraged lay-scientists to develop one action plan to focus on. During the activation meeting, research staff and lay-scientists were unsuccessful in formulating a feasible action plan agreed upon by the group, which led to a post-activation meeting with one clergy member to finalize one priority area to focus on.

Overall, each church developed contextually relevant solutions to promote congregational health, community health, or both. Examples of action plans included: (1) Epidemiological approaches to identify congregation-level health needs; (2) posting evidence-based messaging across the church building (e.g., physical activity and healthy eating recommendations); and (3) activating their social and built environments for both congregation- and community-wide health promotion (e.g., church-sponsored health summits, gardening initiatives with local schools) (Table 5).

**Table 5:** Detailed action plans co-developed and validated by lay-scientists and research staff for all three church settings that participated in the *Our Voice* project.

Church setting	Co-developed action plans
Church #1	Priority 1: Design and implement a church-wide survey in Fall 2024 exploring the health-related needs of the congregation and community (e.g., mental, physical, nutritional, etc...). Priority 2: In 2025, hold a community-wide health fair based on pressing/unmet needs identified in the survey. Priority 3: Begin placing evidence-based health promotion messaging (e.g., food pyramid, physical activity, mental health) around the church facility on bulletin boards, slideshows on TV, etc... Priority 3a: Monthly/Quarterly health messages, based on a certain theme, sent as text messages to the entire congregation.
Church #2	Priority 1: Move forward with community outreach centered around the church garden. (a) Start a conversation with the school administrator/principal to better understand needs and interest in a gardening program/partnership with the church, (b) Talk with local churches in the area that have long term gardening initiatives to better understand how they have managed their gardens for so long, how to get the church involved, etc... and (c) Potentially begin a partnership with Alaska Seeds of Change and see if they have interns or others who need hours/outreach opportunities. Priority 2: In 2025, conduct congregational health needs assessment. (a) Identify method and timeline of delivering surveys to congregation (online and paper versions) and (b) Develop plan to deliver survey and broader project results to congregation, and (c) Host community-wide health fair based on pressing needs identified in survey. Priority 3: In 2025, host a community-wide health fair. (a) Identify pressing needs from congregational health needs assessment, (b) Reach out to community partners and community health organizations about health fair, and (c) Design health fair around pressing community needs.
Church #3	Priority 1: In 2025, pilot a community-wide event addressing whole person health. This event will be in direct response to a community need and will include topics related to physical health, intellectual health, emotional health, relational health, and spiritual health.

#### 3.3.4 Change (action plans and short-term indicators)

To date, lay-scientists from church settings #1 and #2 have continued to convene and implement their action plans. Through follow-up and ongoing updates from clergy, research staff have documented the processes in which action plans have been implemented and shared with the broader congregation.

##### 3.3.4.1 Church setting #1

*Action Plan 1 (Evidence-Based Messaging):* Lay-scientists requested assistance in distributing evidence-based health promotion messaging material across the church facility. Research staff, in collaboration with Alaska Department of Health, compiled health promotion material (e.g., smoking cessation, nutrition, physical activity) for lay-scientists to review and select for dissemination.

*Action Plan 2 (Needs Assessment):* Research staff and lay-scientists collaborated to adapt the Mid-South Congregational Health Survey (37). Once adapted, the project was officially announced to the entire congregation during a Sunday sermon. Church staff implemented and analyzed the assessment without research staff assistance. Overall, 24 congregants participated in the survey, and anxiety/depression/stress, nutrition or physical activity, and substance abuse/addiction were the most frequently reported congregational health needs.

*Action Plan 3 (Church-Sponsored Health Summit):* Lay-scientists piloted a community-wide health summit. Specifically, the group designed a day-long health summit and invited research staff to provide information on the project as well as experts to provide practical tips and strategies about physical activity, nutrition and mental health. A representative from the Alaska Department of Health was present to speak about potential partnerships and collaborations. There were ~30–35 total people were in attendance.

##### 3.3.4.2 Church setting #2

*Action Plan 1 (Community Gardening):* Lay scientists have leveraged their longstanding relationship with a nearby elementary school to engage teachers and students in the process of both planting and harvesting vegetables in the church garden. The 5th grade class (57 students) from the local school, along with church volunteers, teachers/teacher assistants, and parents helped plant and harvest vegetables in the church garden. Lay-scientists noted many of the children had not experienced gardening before and it was beneficial for the group. In total, the group grew 226 pounds of vegetables and shared with a local non-profit, the congregation, and neighbors.

*Action Plan 2 (Needs Assessment):* Research staff and lay-scientists collaborated to review and further adapt the Mid-South Congregational Health Survey (37). The project was announced during a Sunday sermon along with encouragement for congregation members to participate in the health needs assessment. Needs assessment findings are still being compiled with plans to host a community-wide health fair to address the top needs identified. Although findings are still being analyzed, the clergy member has used survey findings as well as conversations from community meetings to begin addressing pressing needs (e.g., inviting health/nutrition coaches to speak to the congregation).

##### 3.3.4.3 Church setting #3

Lay-scientists did not continue to meet, although follow-up conversations with clergy indicated plans to implement a community-wide event addressing whole person health are underway. There are some lessons learned with this particular site. Specifically, the compressed format, the numerous competing demands, use of their space and the capacity concerns of the clergy member, differences in community priorities between clergy and congregants all likely influenced their readiness to formulate an action agreed upon by the group.

### 3.4 Pre-post survey comparisons

For the social cohesion and agency for scale a significant increase was seen in one item (“By working together with others in my church, we can influence decisions that affect our church.”) from baseline to follow-up (n = 14; pre: 4.21 ± 0.80; post: 4.50 ± 0.80; *Z* = −2.0; *p* = 0.046). Across the three church settings, no statistically significant pre-post changes were seen in physical activity or healthy eating self-efficacy (all *p-*values > 0.05) (Table 6).

**Table 6:** Cross-sample comparisons of pre- and post-survey outcomes among lay-scientists who participated in an *Our Voice* pilot study (*n* = 14).

Variable	Pre-survey mean (SD)	Post-survey mean (SD)	*Z*-score	*p*-value
Physical activity self-efficacy (PASE)	83.6 (18.2)	83.8 (21.8)	−0.5	0.634
Healthy eating self-efficacy (HESE)	79.2 (19.1)	81.6 (14.3)	−0.4	0.687
Social cohesion and agency				
*“I feel safe in my church”*	4.9 (0.4)	4.5 (1.1)	−1.1	0.257
*“This is a church where people support each other”*	4.6 (0.5)	4.1 (1.1)	−1.1	0.276
*“I can influence decisions that affect my church”*	4.1 (1.0)	4.3 (0.8)	−1.1	0.257
*“Decision-makers in this church care about the opinions and experiences of church members”*	4.3 (1.0)	4.6 (0.6)	−1.9	0.059
*“By working together with others in my church, we can influence decisions that affect our church”*	4.2 (0.8)	4.5 (0.7)	−2.0	**0.046**
*“People in my church know who to talk to in order to make changes happen in our church”*	4.1 (0.8)	4.4 (0.8)	−1.3	0.180
*“My pastor should have time for their own self-care”*	4.9 (0.5)	4.8 (0.6)	−1.0	0.317

Three participants excluded from analysis due to incomplete follow-up survey. Bold values indicate significance at *p* < 0.05.

## 4 Discussion

In this first-generation study, we piloted the *Our Voice* method (22, 23) with three Alaskan churches differing in size, location, and congregant demographics. Prior to study activities, research staff incorporated feedback from local community experts, public health officials, and academic researchers to maximize the reach, effectiveness, and potential sustainability of *Our Voice* within faith-based settings. Over a year, project staff conducted three church environmental audits to assess the health promotion opportunities within and around each church setting. Activated lay-scientists (clergy and congregants) engaged in lived experience data collection, identified contextually relevant solutions and co-designed action plans to leverage the social and built environments of their church to increase opportunities for health promotion in the congregations and communities they serve. This study provides preliminary evidence of the applicability of *Our Voice* within faith-based settings and how it can serve as a catalyst to impact not only clergy and congregational health, but also potentially the broader communities they serve. Study findings also provide insight into how church size, church space constraints, capacity of clergy, and differing perceptions of congregational health priorities can influence the development and implementation of action plans.

As described earlier, the study protocol focused on using both the structured researcher-collected church environmental audit tool (36) and the citizen scientist Discovery Tool (24) to document the health promotion opportunities within each church setting. The “by the people” nature of *Our Voice* brought to light the necessity of bringing diverse perspectives to the table when preparing and implementing community-level interventions (20). For example, the project revealed that each church setting had contextually relevant opportunities and solutions for health promotion, as evident in the audit tool findings as well as the Discovery Tool photo and narrative data. This is also consistent with previous literature that highlights how different communities and settings, particularly churches, may have differing priorities and needs, which needs to be considered when planning health promotion programs (5). Study findings revealed that the *Our Voice* process, specifically, encouraged activated lay-scientists to identify and develop contextually relevant solutions based on feasibility of implementation and existing resources within church settings, particularly within church #1 (e.g., distributing evidence-based materials across church facility) and #2 (engaging local school in community gardening initiatives).

The lived experience data collection and community meetings process also provided greater context and insights into how external and internal factors can impede health promotion opportunities within church settings. For example, when considering church #3, a small, rural church, the researcher-led church audit identified many opportunities for health promotion (e.g., half-size gym, kitchen); yet through the Discovery Tool and community meetings, lay-scientists (i.e., community experts) provided in-depth insight into church space constraints. One major barrier described was that an outside organization occupied some of the main areas that potentially could be leveraged for both congregation- and broader community-level health promotion. It should be noted that while previous literature has shown churches greater in size are likely to have more resources for health promotion programming (38), barriers for utilizing church space for health-related activities has been documented even among larger churches (39). Thus, future *Our Voice* research within faith-based settings should continue conducting researcher-led environmental assessments to complement the participant-led data collection; use of validated instruments such as the EAT, as well as assessing other relevant data (e.g., communication technology used by the church) may also help inform the design of *Our Voice* logistics and protocols as well as to encourage lay-scientists to maximize their church’s space and resources when co-developing solutions for health promotion.

This pilot study also provided insight into the importance of having a rigorous, yet flexible research protocol to ensure researcher and community priorities align (40, 41). This was particularly the case in focusing on physical activity and healthy eating while also accounting for mental and spiritual health (39, 42). While the community meetings did have a focus on physical and nutritional health, the project focus shift over time to holistic wellbeing may explain why no pre-post changes were seen in exercise and diet self-efficacy across the reasonably short study time period. Such findings may also indicate that participants may additionally benefit when it comes to self-efficacy by including additional “person-level” exercise and dietary behavior change strategies that specifically target the varied personal barriers that often exist when changing these health behaviors (43–45). Nonetheless, this study provides preliminary evidence that future *Our Voice* initiatives, particularly in faith-based settings, should continue incorporating a holistic focus and unpacking the connection between physical, mental, and spiritual health (39, 42) and what that means for individual- and community-level behavior change. Future research is also needed to continue examining how action plans generated by lay-scientists through the *Our Voice* process may impact health and behavior of the congregation and broader community over time.

It is also well documented that lived experience data collected throughout the *Our Voice* process can be used to successfully advocate for changes in local environment and policies in collaboration with decision-makers and key community stakeholders (46). In this study, the key decision-makers (i.e., clergy) played an essential role in allowing research to commence within their faith community, assisting in recruiting representative members to participate, and documenting, identifying, and develop action-oriented solutions. This finding aligns with previous research showing clergy buy-in, and even participation, is essential for the successful implementation of faith-based health promotion programming within the congregations they serve (11).

Study findings showed that through the *Our Voice* process clergy and congregants can work together, synthesize data, and identify and implement data-driven solutions to address pressing priorities. This action-oriented collaboration between lay-scientists may explain the pre-post changes seen in social cohesion and agency (i.e., “By working together with others in my church, we can influence decisions that affect our church.”). Significant pre-post changes in social/neighborhood cohesion have also been seen in previous *Our Voice* research aimed at promoting and advocating for inclusive environments as well as identifying barriers to active living (19, 23). Future research implementing the *Our Voice* method within faith-based settings may benefit from incorporating “post-journey” interviews (47, 48) with lay-scientists to gain deeper insight into their experiences and understand the context around factors impacting cohesion and collective agency.

Previous research has also documented that clergy and congregants within church settings may perceive community health needs and priorities differently (16, 49), which was also highlighted through photos and narratives collected through the Discovery Tool as well as across community meetings, and may serve as a barrier when developing feasible solutions. For example, within church setting #3, we found that discordant perceptions of priorities between clergy and congregants, along with space constraints, made it difficult to co-develop and implement a feasible action plan addressing identified priorities. Future *Our Voice* studies within faith-based settings should assess lay-scientist knowledge and attitudes towards health behavior (e.g., physical activity, healthy eating) as well as the role churches can play in health promotion. Collecting individual-level health perceptions as well as overall church characteristics (e.g., church size, staff support, existing resources) may provide a more well-rounded picture of internal and external factors potentially influencing the development, implementation and sustainability of health promotion programming within and across faith-based settings.

While clergy serve as gatekeepers for health promotion, they often report a high prevalence of poor health outcomes (12); unfortunately, their health is often overlooked in congregational health promotion programming (14), even though churches serve as clergy worksites. Recent evidence suggests congregation-level adaptations (i.e., creating physical activity and healthy eating opportunities, gaining clergy support for health programming) may have long-term, indirect effects on clergy health (14). For example, the Faith Activity Nutrition program (FAN) found that despite focusing on congregational member diet and physical activity behaviors, at the end of 12 months clergy physical activity engagement also increased (i.e., meeting the physical activity guidelines) (14). Although clergy were directly involved in the planning and implementation of action plans to address congregational health priorities, our short follow-up time as well as expanded focus on holistic health (physical, nutritional, mental, spiritual) made it difficult to assess project impact on clergy health outcomes. The next phase of *Our Voice* research within faith-based settings may benefit from conducting pre- and post- qualitative interviews with participating clergy to gain in-depth insight into how direct involvement in the adaptation of their church’s social and built environment (i.e., clergy worksites) can support or hinder individual-level health and behavior outcomes.

### 4.1 Strengths

This study has several strengths. We engaged community “experts” throughout the entirety of the project, which enhanced our ability to ensure this project was contextually relevant as well as faith-based and not simply “faith-placed” (i.e., standard intervention implemented in faith setting without community input) (5). Applying the *Our Voice* method was a strength in that it allowed lay-scientists to collect their own data, lead community conversations, and begin designing community-aligned strategies to implement developed action plans. Our methodology also allowed for the implementation of a core standardized, yet adaptable method across church settings, but paved the way for each setting to identify contextually-relevant and customized priorities. Another strength was incorporating both diverse clergy and congregants across race/ethnicity, and church size in the *Our Voice* process, which allowed us to better understand how different groups of community members and stakeholders could participate in and contribute to the development of community level changes.

### 4.2 Limitations

As with any study, there were limitations. Each participating church was in Alaska, which may make our findings difficult to generalize to other churches across the US. Future research should build upon this model with other churches to identify similarities and differences in implementation. However, our purposeful selection of three churches that varied greatly in size, congregant demographics, and urban, suburban, rural communities provided an opportunity to explore differences in logistical approaches, priorities, and capacity to develop and implement feasible action plans. The small number of participating lay-scientists recommended by clergy across church settings may have introduced selection bias and not have allowed us to fully identify pressing needs in the community as they may not have representative of the broader congregation. With that said, lay-scientists that were recommended by clergy and that did participate had high engagement throughout the process. The short duration to follow-up coupled with the sample size may have impacted our ability to detect differences in collective agency and social cohesion as well as physical activity and diet self-efficacy across church settings. Further investigations with larger samples and longer time periods can help to shed further light on the issues identified in this first-generation study.

## 5 Conclusion

*Our Voice* studies have shown that engaging community members in a lived experience data collection process can lead to meaningful changes to local social and built environments (19, 21, 23, 25). Like any community organization, churches have unique environments, cultural traditions, and needs; yet churches are also recognized as important community settings that can help to advance public health messaging and community needs. This community-engaged pilot study provides a potential framework for academic-faith community partnerships when co-designing community-aligned, faith-based interventions. Future research using longitudinal designs with larger, more diverse samples is needed to provide more actionable health promotion insights and better assess the effectiveness of the *Our Voice* method in faith-based settings.

## Data Availability

The datasets presented in this article are not readily available due to the small sample size and potentially identifiable Discovery Tool photos and narratives (stored in a secure server). Reasonable requests to access the datasets should be directed to ntwest@alaska.edu.
